# Chronic Serpiginous Ulcer of the Cornea

**Published:** 1901-11-23

**Authors:** 


### CHRONIC SERPIGINOUS ULCER OF THE CORNEA.

The principal subject of discussion at the last meeting of the Ophthalmological Society was "Chronic Serpiginous Ulceration of the Cornea," commonly known also under the name of "Mooren's ulcer." Mr. E. Nettleship, who read a paper based upon an examination of 71 such cases, 12 of which had been under his care, said that the usual duration of the disease had been from 4 to 12 months, and the ages of the subjects ranged from 23 to 71 years. In a large majority of the cases the attack began in the winter half of the year. The course of the disease strongly suggested infection as a cause, but no special micro-organism had yet been found. In more than a fourth of the cases both eyes suffered, although sometimes at an interval of years. The prognosis was always grave, and was far worse when both eyes were attacked, only one in four of the double cases being arrested short of total leucoma, while of the single cases more than one half recovered with some untouched cornea. In this disease the deeper parts of the eye remain healthy, and the amount of vision left is determined by the final state of the cornea and pupil. In regard to treatment the first thing to do is to cut away the overhanging and half-dead edge of the ulcer and apply an escharotic or strong germicide to its advancing border thus laid bare. Mr. Nettleship thought that the galvano-cautery was the best, while pure carbolic acid and strong tincture of iodine probably came next. Transplantation of a conjunctival flap over the ulcer appeared useful occasionally.

